# The relationship between hyperuricemia and contrast-induced acute kidney injury undergoing primary percutaneous coronary intervention: secondary analysis protocol for the ATTEMPT RESCIND-1 study

**DOI:** 10.1186/s13063-020-04505-w

**Published:** 2020-06-24

**Authors:** Wei Guo, Feier Song, Shiqun Chen, Li Zhang, Guoli Sun, Jin Liu, Jiyan Chen, Yong Liu, Ning Tan, the RESCIND group

**Affiliations:** 1 Department of Cardiology, Guangdong Provincial Key Laboratory of Coronary Heart Disease Prevention, Guangdong Cardiovascular Institute, Guangdong Provincial People’s Hospital, Guangdong Academy of Medical Sciences, Guangzhou, 510080 China; 2 Guangdong Provincial Geriatrics Institute, Guangdong Provincial People’s Hospital, Guangdong Academy of Medical Sciences, Guangzhou, 510100 China

**Keywords:** Hyperuricemia, Contrast-induced acute kidney, ST-segment elevation myocardial infarction, Primary percutaneous coronary intervention

## Abstract

**Background:**

Contrast-induced acute kidney injury (CI-AKI) contributes toward unfavorable clinical outcomes after primary percutaneous coronary intervention (pPCI). We will assess whether hyperuricemia is an independent predictor of CI-AKI and outcomes in patients undergoing pPCI.

**Methods/design:**

Our study is a secondary analysis for the database from ATTEMPT study, enrolling 560 ST-segment elevation myocardial infarction (STEMI) patients undergoing pPCI. Patients will be divided into 2 groups according to the admission serum uric acid (SUA) level. Hyperuricemia will be defined as a SUA level > 7 mg/dL (417 mmol/L) in males and > 6 mg/dL (357 mmol/L) in females. The primary endpoint was CI-AKI, defined as > 25% or 0.5 mg/dL increase in serum creatinine from baseline during the first 48–72 h post-procedurally. Multivariate analyses for CI-AKI and long-term mortality will be performed using the logistic regression and Cox regression analyses, respectively.

**Discussion:**

This study will determine the predictive value of hyperuricemia for the development of CI-AKI and outcomes in patients with STEMI undergoing pPCI. We predict that hyperuricemia will be associated with a risk of CI-AKI in patients with pPCI. Furthermore, after adjusting for other variables, long-term mortality after pPCI may be higher in those with hyperuricemia than in those with normouricemia. Results of this study may provide scientific evidence for the effect of hyperuricemia on CI-AKI and long-term outcomes, thereby offering the potential possibility of lowering SUA on the development of CI-AKI and outcomes.

**Trial registration:**

ClinicalTrials.gov NCT02067195, Registered on 20 February 2014.

## Background

ST-elevation myocardial infarction (STEMI) patients undergoing primary percutaneous coronary intervention (pPCI) are commonly complicated by contrast-induced acute kidney injury (CI-AKI) [1]. CI-AKI is associated with higher hospitalization rates, long-term morbidity, and mortality [2]. Therefore, patients recognized with high-risk factors of CI-AKI should be carefully monitored and treated with appropriate prophylactic strategies. Based on previous study, chronic kidney disease, diabetes, hypotension, contrast volume, congestive heart failure, advanced age, and anemia have been identified as risk factors of CI-AKI [3].

Uric acid is the final product of purine metabolism, which is metabolized by xanthine oxidase [4]. Substantial evidence suggests that hyperuricemia is an independent risk factor for hypertension, metabolic syndrome, chronic kidney disease, and cardiovascular diseases [5]. In previous studies, we reported that hyperuricemia was an independent risk factor for CI-AKI after PCI [6, 7]. However, the conclusion remains controversial [8–15]. In addition, only a limited number of studies reported that hyperuricemia was an independent predictor of CI-AKI in STEMI patients undergoing pPCI. Moreover, it was uncertain about the role of uric acid in the long-term outcome [16–18].

We aimed to investigate the association of hyperuricemia with CI-AKI in patients with high-risk STEMI undergoing pPCI and to determine the predicting role in prognosis.

## Methods/design

### Study design and population

This is a secondary analysis of aggressive hydration in patients with STEMI undergoing pPCI to prevent contrast-induced nephropathy, the first study for reduction of contrast-induced nephropathy following cardiac catheterization (ATTEMPT RESCIND-1 study). Five hundred sixty patients aged 18 years or older, treated with pPCI, and provided written informed consent were included from 15 medical research centers in China. Inclusion and exclusion criteria were described elsewhere [19]. We also excluded the patients without the admission serum uric acid (SUA) level.

### Study protocol

Baseline data will include demographics, diagnosis, medical history, laboratory parameters, medications, and physical examination. Patients will be divided into 2 groups according to the admission serum uric acid (SUA) level. Hyperuricemia will be defined as a SUA level > 7 mg/dL (417 mmol/L) in males and > 6 mg/dL (357 mmol/L) in females. The comorbidities will include hypertension, diabetes mellitus, hyperlipidemia, heart failure, chronic kidney disease, stroke, peripheral arterial disease, and chronic obstructive pulmonary disease. The concomitant therapies were decided by the doctors and were relied on the routine standards of care recommended by the current guidelines. The primary outcome, CI-AKI, was defined as a 25% or 0.5 mg/dL increase in serum creatinine from baseline at 48–72 h after pPCI [19]. The secondary endpoints were different definitions of CI-AKI, persistent renal impairment, major adverse clinical events during hospitalization, total hospitalization costs, and length of hospital stay [19].

Follow-up adverse events will be recorded by trained investigators via office visits or telephone interviews at 3, 6, 12, 18, and 24 months after the pPCI. Long-term outcomes will be major adverse cardiovascular events (MACEs), including mortality, stent restenosis, non-fatal myocardial infarction, and target vessel revascularization.

All data will be collected with standardized electronic case report forms. At the time of enrollment, the data management team of Guangdong Provincial People’s Hospital will conduct consistency checks and issues data clarification forms to deal with discrepant data. All data will be collected after the approval by the ethics committee of all participating centers. An independent data monitoring committee will review the ongoing safety events of every participant.

How data are stored and managed had explained in the “data and safety monitoring board” of our previous study [19]. The details are as follows. A committee of clinicians and a biostatistician, chaired by Chun Wang, MD, and Chun-quan Ou, MS, who are blinded to the treatment allocation, will periodically review and evaluate the accumulated study data for participant safety, study conduct, and progress, and, when appropriate, efficacy and will make recommendations to the principal investigators concerning the continuation, modification of enrollment, or termination of the trial. Details of any protocol violations (e.g., descriptions, reasons, and resolution) will be reported by the investigators and clinical research associates to the primary investigators, the Central Event Adjudication committee, and Data and Safety Monitoring Board, if necessary. In addition, training will be provided to the researchers, and the study procedure will be supervised, with particular attention to the reasons for bias and data verification to avoid recurrence of protocol violations. Finally, intention-to-treat analysis will be used for the patients with protocol violations instead of the per-protocol analysis [19].

The literature review of hyperuricemia and CI-AKI has reported to the RESCIND group’s academic committee. After that, a statistical scheme will be written and submitted to statisticians and data administrators to obtain data.

### Statistical analysis

Baseline patient characteristics and information will be presented as the mean ± standard deviation or median plus interquartile ranges of descriptive statistics. They will be compared by *t* test or Wilcoxon rank sum test according to distribution. The categorical data about baseline characteristics and information will be expressed as a percentage were analyzed using the Pearson chi-square test or Fisher’s exact test. The association between hyperuricemia and CI-AKI will be performed using multivariate logistic regression analysis. Multivariable logistic regression models have been developed to adjust for clinical characteristics (e.g., age, sex, estimated glomerular filtration rate, left ventricular ejection fraction, chronic heart failure, anemia, diabetes mellitus), and the Mehran risk score will be calculated [3]. All variables that are univariately associated with this outcome measure will be entered as possible predictors in a multivariable logistic regression analysis. The association between hyperuricemia with long-term mortality will be investigated by Cox regression model adjusting for other risk factors (e.g., age, CI-AKI, heart function, renal function, and medication). Kaplan-Meier curve and log-rank test will be performed on the survival time of the hyperuricemia group and the normouricemia group (the cumulative rate of all-cause mortality and MACEs between these two groups). These data will be analyzed on the basis of valid cases. A two-tailed *p* value < 0.05 will be considered statistically significant. The power calculation was based on our previous finding [20], and a CI-AKI incidence of 23% was estimated for the hyperuricemia group, while 11.5% was assumed for the normouricemia group (50% relative reduction). Using a two-sided chi-square test, a significance level of 0.05, and a sample size of 280 each group, the power of the tests is over 90%.

All statistical analyses will be performed using SAS version 9.4 or later (SAS Institute, Cary, NC, USA) and R software (version 3.1.2; R Foundation for Statistical Computing, Vienna, Austria).

## Discussion

CI-AKI is closely associated with prolonged hospital stay, long-term morbidity, and mortality in patient undergoing PCI. The incidence of CI-AKI is about 2% in the general population and over 50% in high-risk population [3]. Previous study supported the relationship between CI-AKI and higher incidence of adverse short- and long-term cardiovascular outcomes, including mortality [21]. Patients with STEMI are likely to present with hypotension, or even cardiogenic shock, higher volume of contrast media, and impossibility of renal prophylactic therapy, which are associated with an increased risk of CI-AKI [22].

Advanced age, diabetes, dehydration, hypotension, sepsis, cardiovascular disease, underlying acute kidney injury, chronic kidney disease, and concomitant use of nephrotoxic drugs were identified as well-known risk factors for CI-AKI [3, 23]. In previous studies [6, 7], we reported that hyperuricemia was an independent risk factor for CI-AKI in PCI patients, which was consistent with other studies [8–13]. However, other studies did not show the same conclusion [14, 15]. Several studies explored the effect of serum uric acid (SUA) on CI-AKI among high-risk patients such as STEMI undergoing primary PCI. Elbasan et al. showed that SUA was associated with CI-AKI in STEMI patients undergoing pPCI (mean SUA = 6.2 ± 0.9 mg/dL, 95% CI, 1.877–3.236; *p* = 0.002) [16]. In another study, Mendi et al. demonstrated that SUA ≥ 5.4 mg/dL was an independent risk factor for CI-AKI (OR 1.26, 95% CI, 1.10–1.42; *p* < 0.001) [17]. Saritemur et al. showed that elevated uric acid was lined with CI-AKI in multivariate analysis after adjusting for potential confounding factors (OR 1.01, 95% CI, 1.00–1.01; *p* = 0.01) [18]. However, even though they reported that high SUA was an independent predictor of CI-AKI in STEMI patients, there was no uniform standard for the definition of hyperuricemia. In our study, we will employ the definition consistent with our previous study.

In addition, these studies did not compare the prognosis between CI-AKI group and non-CI-AKI group in STEMI patients undergoing PCI. Some observational studies proved the relationship between hyperuricemia and clinical outcomes in the presence of gout, but not for asymptomatic hyperuricemia [24, 25]. Nevertheless, a recent study by Pagidipati et al., including the PLATO and TRACER study population, demonstrated a significant association between uric acid (UA) and short-term adverse outcomes, independent of the presence of gout [26]. Regarding the relationship between UA and long-term prognosis, the data also supported the relationship between elevated UA and long-term prognosis in patient with acute coronary syndromes and treated with PCI [27]. Therefore, whether hyperuricemia is still a predictor of poor long-term prognosis after adjusting for CI-AKI should be studied in STEMI patients receiving pPCI.

Although the pathophysiological mechanisms of adverse reactions to hyperuricemia have not been fully elucidated, it appears to be multifactorial. In experimental models, hyperuricemia was linked to a variety of proatherogenic processes, including increased oxidative stress [28], vascular smooth muscle cell proliferation [29], inflammation [30], and endothelial dysfunction [31].

### Limitations

Our current secondary analysis is subject to the following restrictions. First, its sensitivity was lower due to the definition of > 0.5 mg/dL because it was less selective for patients with higher risk of mortality and morbidity. Second, single baseline SUA measurements were used to predict CI-AKI and long-term mortality. However, a number of previous studies have used this method as well. Third, the measurement of serum creatinine (SCr) was standardized at 72 h after pPCI rather than at random, which might lead to the ignorance at the increase in delayed SCr (> 72 h). Finally, it is a secondary analysis that is not capable of testifying a causal relationship.

In conclusion, our study will determine the association between hyperuricemia and contrast-induced acute kidney injury (CI-AKI) after primary percutaneous coronary intervention (pPCI) and will identify if hyperuricemia is a risk factor for long-term death after adjusting for CI-AKI.

### Trial status

The first patient was included in the RESCIND –1 ATTEMPT Trial (Protocol version 2.0, 11 June 2014) on 1 July 2014 and expected to complete recruitment in December 2018. As of October 24, 2018, recruitment is ongoing with 555 patients randomized at 15 centers in China.

## Data Availability

Data sharing not applicable to this article as no datasets were generated or analyzed during the current study.

## Abbreviations

CI-AKI: Contrast-induced acute kidney injury
pPCI: Primary percutaneous coronary intervention
STEMI: ST-segment elevation myocardial infarction
SUA: Serum uric acid
SCr: Serum creatinine
